# Equine Rotavirus A under the One Health Lens: Potential Impacts on Public Health

**DOI:** 10.3390/v16010130

**Published:** 2024-01-16

**Authors:** Mariano Carossino, Maria Aldana Vissani, Maria E. Barrandeguy, Udeni B. R. Balasuriya, Viviana Parreño

**Affiliations:** 1 Department of Pathobiological Sciences, School of Veterinary Medicine, Louisiana State University, Baton Rouge, LA 70803, USA; balasuriya1@lsu.edu; 2 Louisiana Animal Disease Diagnostic Laboratory, School of Veterinary Medicine, Louisiana State University, Baton Rouge, LA 70803, USA; 3 Escuela de Veterinaria, Facultad de Ciencias Agrarias y Veterinarias, Universidad del Salvador, Pilar, Buenos Aires B1630AHU, Argentina; vissani.aldana@inta.gob.ar (M.A.V.); maria.barrandeguy@usal.edu.ar (M.E.B.); 4 Instituto de Virología, CICVyA, Instituto Nacional de Tecnología Agropecuaria (INTA), Buenos Aires B1686LQF, Argentina; parreno.viviana@inta.gob.ar; 5 Consejo Nacional de Investigaciones Científicas y Técnicas (CONICET), Buenos Aires C1033AAJ, Argentina

**Keywords:** rotavirus, group A rotavirus, equine rotavirus A, One Health, public health, diarrhea, reassortment, animal–human transmission, zoonosis, equine-like G3, rotavirus vaccine

## Abstract

Group A rotaviruses are a well-known cause of viral gastroenteritis in infants and children, as well as in many mammalian species and birds, affecting them at a young age. This group of viruses has a double-stranded, segmented RNA genome with high genetic diversity linked to point mutations, recombination, and, importantly, reassortment. While initial molecular investigations undertaken in the 1900s suggested host range restriction among group A rotaviruses based on the fact that different gene segments were distributed among different animal species, recent molecular surveillance and genome constellation genotyping studies conducted by the Rotavirus Classification Working Group (RCWG) have shown that animal rotaviruses serve as a source of diversification of human rotavirus A, highlighting their zoonotic potential. Rotaviruses occurring in various animal species have been linked with contributing genetic material to human rotaviruses, including horses, with the most recent identification of equine-like G3 rotavirus A infecting children. The goal of this article is to review relevant information related to rotavirus structure/genomic organization, epidemiology (with a focus on human and equine rotavirus A), evolution, inter-species transmission, and the potential zoonotic role of equine and other animal rotaviruses. Diagnostics, surveillance and the current status of human and livestock vaccines against RVA are also reviewed.

## 1. Introduction

Group A rotaviruses (RVA) are a group of enteric viruses responsible for acute gastroenteritis and watery diarrhea that affect infant children, numerous young mammals, and birds [1,2,3]. Prior to their recognition as a major cause of gastroenteritis in infant children in 1973 [4], rotaviruses have already been identified as a cause of diarrhea in several animal species including mice, monkeys, pigs, cattle, cats, and dogs [5,6,7,8,9]. Currently, human rotaviral infections account for >600,000 child deaths annually, particularly in developing countries [10], and have been identified as a major veterinary pathogen with significant impact to agricultural production systems, particularly involving young calves, weaning and post-weaning piglets, and foals [1,11,12,13]. 

Rotaviruses are segmented, double-stranded RNA viruses belonging to the family *Sedoreoviridae* (formerly *Reoviridae*) [3]. While genetically diverse, host range restriction has been suggested based on specific gene segment distribution across animal species [2]. However, more recent molecular epidemiology studies on both humans and animal rotaviruses have shown that animal rotaviruses can serve as a source of genetic material contributing to human rotavirus diversity [2,11,14]. In recent years, an equine-like G3 rotavirus A affecting children has been identified as an emergent virus [15], demonstrating that equine rotaviruses can also serve as a source of such genetic diversification and pose zoonotic potential. Herein, various aspects of rotavirus biology, cross-species transmission, and zoonotic potential are reviewed, with a specific emphasis on equine rotaviruses and their relevance from a One Health perspective. We also review aspects related to diagnostics, surveillance, and vaccines for human and livestock use. 

## 2. Structure, Genome Organization, Classification, and Biology of Rotaviruses

**Figure 1:**
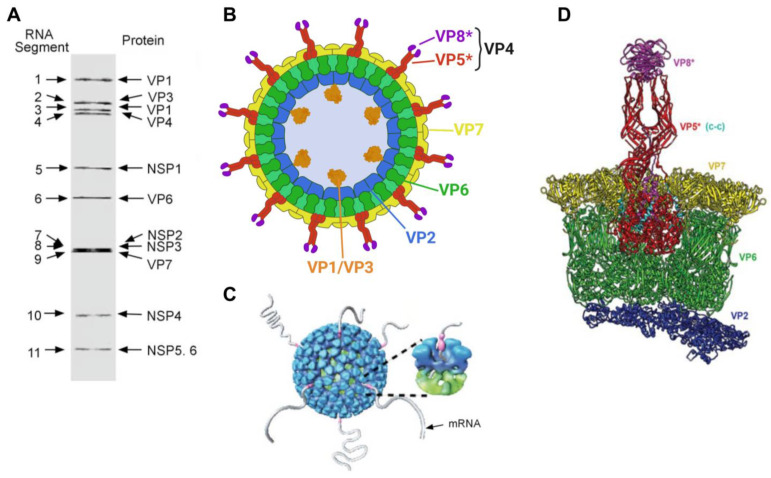
Rotavirus structure: (**A**) Polyacrylamide gel electrophoresis (PAGE) analysis showing 11 dsRNA genomic segments of RVA. Gene segments and encoded proteins are numbered to the left and to the right, respectively. (**B**) Schematic diagram of the rotavirus particle. The infectious triple-layered particle (TLP) comprises an outer capsid composed of VP7 (yellow) and VP4 (red/purple) spikes, a middle layer composed of VP6 (green), and an inner layer composed of VP2 (blue) to which transcriptional enzymes VP1 and VP3 are anchored (orange). (**C**) Cryo-EM reconstruction of transcribing double-layered particles (DLP). The endogenous transcription results in the simultaneous release of the transcribed mRNAs from channels located at the five-fold vertices of the icosahedral DLP. Reprinted from Jayaram et al. Emerging themes in rotavirus cell entry, genome organization, transcription, and replication. *Virus Res*. 2004 Apr;101(1):67-81 with permission [16]. (**D**) Reconstruction of the interacting viral proteins composing the TLP. The VP4 spike (VP8* in magenta, VP5* in red, and the VP5* segment that will form the coiled coil in the ‘post-entry’ conformation in cyan) has globular foot domains on VP5* anchored in the VP6 layer (green). The VP6 lattice overlays the VP2 inner core shell (dark blue). The VP7 trimer (yellow) caps each trimer of VP6. The viral genome is contained within the VP2 inner core shell (not shown). Reprinted from Settembre et al., Atomic model of an infectious rotavirus particle. *EMBO J*. 2011 Jan 19;30(2):408-16 with permission [22].

**Figure 2:**
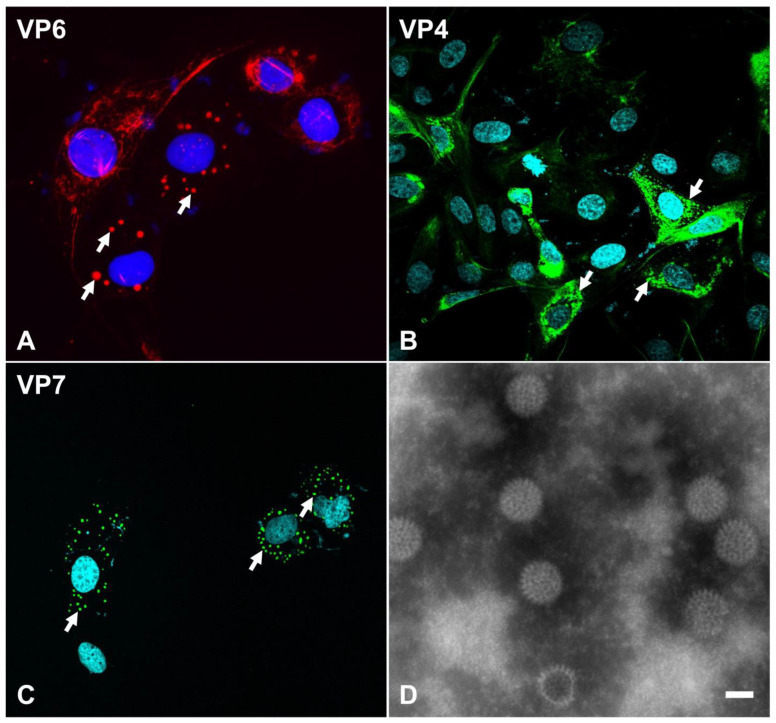
Expression of rotavirus structural proteins in infected cells (**A**–**C**) and morphological features of the infectious virus particle under transmission electron microscopy (**D**). MA-104 cells were infected with the equine RVA H2 G3P[12] strain and immunolabeled using antibodies specific to VP6 ((**A**), AlexaFluor^®^ 594), equine RVA VP4 ((**B**), AlexaFluor^®^ 488), or equine RVA VP7 ((**C**), AlexaFluor^®^ 488). Specifically labeled viral structural proteins are noted within viroplasms in the cellular cytoplasm (arrows). (**D**) Negative staining of equine RVA particles following virus purification from tissue culture supernatant. RVA particles have a typical “wheel” shape with spike-like projections from the outer capsid. Transmission electron microscopy, 40,000× magnification. Bar = 50 μm. This image is reprinted with permission (Carossino et al., Detection, molecular characterization and phylogenetic analysis of G3P[12] and G14P[12] equine rotavirus strains co-circulating in central Kentucky, *Virus Res* 2018 Vol. 255 Pages 39–54 [48]).

**Structure.** In over 50 years since their identification as a cause of infant gastroenteritis in humans, the structure and composition of the rotavirus particle has been thoroughly characterized ([4,16,17,18,19] and references therein). The rotavirus particle is structurally complex, with the fully infectious rotavirus particle being non-enveloped and composed of three concentric protein layers measuring approximately 100 nm in diameter (also known as a triple-layered particle (TLP)). Under negative-contrast electron microscopy, rotaviral particles have a “wheel-like” appearance, and such a shape has coined “*Rotavirus*” (from the latin rota = wheel) as the accepted genus name (summarized by [3]) (Figure 1 and Figure 2). The TLP encapsidates the viral genome, which is composed of 11 segments of double-stranded RNA (dsRNA, see below for additional details). The composition of the viral particle and genomic arrangement are summarized in Table 1 and Figure 1 and Figure 2. The inner-most layer (single layered particle (SLP), core shell or inner capsid) of the viral particle is composed of 120 copies of viral protein 2 (VP2) arranged as 12 decamers with 12, 5-fold vertices forming an icosahedral core particle of 50–60 nm in diameter. The SLP contains the 11 genomic dsRNA segments and the inner surface of the VP2 core shell at each of the vertices tethers a single polymerase complex composed of the viral RNA-dependent RNA polymerase (RdRp, also known as VP1) and the viral RNA-capping enzyme (VP3). Each genome segment is dedicated to a single polymerase complex, which mediates endogenous transcription within the inner core shell upon cell infection [20]. The core shell is surrounded by 260 trimers of the viral structural protein VP6, which form the middle layer or intermediate capsid. The VP6 trimers are in close contact with both the underlying VP2 as well as the viral structural proteins VP7 and VP4 on the outside layer. The non-infectious particles composed of the inner core shell and the middle layer (VP6) are known as double-layered particles (DLPs). At each one of the five-fold vertices, both the VP2 and VP6 are traversed by an aqueous channel that allows the release of positive-stranded RNA generated during the transcription process (viral mRNA) and the entry of nucleotide triphosphates and divalent cations required by the polymerase complex within the inner core shell (Figure 1) [20]. Finally, DLPs are covered by an outer capsid layer composed of 260 trimers of VP7 making up the outermost shell, and 60 spikes of VP4 trimers that project from the outer VP7 layer to constitute the TLPs [21,22]. Trypsin (or trypsin-like protease)-mediated cleavage of VP4 generates two non-covalently bound subunits, the amino-terminal VP8* and the carboxyl-terminal VP5 subunits, enhancing infectivity [23,24,25]. The VP8* subunit contains the receptor binding domain and is supported by VP5*, which widely interacts with the VP6 within the middle layer (Figure 1). Conformational changes following cleavage of VP4 are critical for penetration of the host’s cell membrane [22,25,26,27,28]. Importantly, an intact outer VP7–VP4 layer is critical for TLP infectivity, and such integrity is dependent on Ca^2+^ ions [29]. Thus, treatment of TLPs with Ca^2+^ chelating agents such as ethylenediaminetetraacetic acid (EDTA) [30] induces destabilization of VP7 trimers, outer layer disassembly, and formation of non-infectious DLPs. DLPs can be subsequently converted to SLPs by chaotropic agents such as sodium thiocyanate or high concentrations of CaCl_2_ [29,31].

**Genomic organization.** The rotavirus genome is composed of 11 (fully base-paired) dsRNA segments (Table 1 and Figure 1). The simian RVA strain SA11 is regarded as the prototype RVA strain (GenBank accessions NC_011500-NC_011510), and its total genome size is 18,555 bp, with individual segments ranging from 667 bp to 3302 bp [32]. Comparatively, the total genome of the reference equine RVA strain H2 is 18,479 bp, with individual segments ranging from 666 bp to 3302 bp (GenBank accessions KM454492.1-KM454502.1). All genome segments share a conserved 5′ and 3′ terminal sequence, followed by a second 30–40 bp, conserved, segment-specific sequence [3,33,34]. Thus, differences at the 3′ end of the dsRNA segments between different rotavirus species are considered to be the reason for the lack of reassortment between rotavirus species [35]. The RVA genome encodes for 13 viral proteins, including 6–7 structural proteins (VP1–VP4, VP6, and VP7 (with two initiation codons on segment 9 that yield two overlapping forms of VP7)) and six nonstructural proteins (NSP1-NSP6) [3,17,36]. Each genome segment is monocistronic and encodes for a single viral protein except for segment 11, which contains two out-of-frame ORFs that encode NSP5 and NSP6 [3].

**Classification.** Rotaviruses are currently classified within a single genus (*Rotavirus*) in the family *Sedoreoviridae*, order *Reovirales*, among five other genera within this family [3]. Members of the genus *Rotavirus* are currently further grouped into 11 different species (Rotavirus A, B, C, D, F, G, H, I, J, K, and L), and such classification is supported based on VP6 identification with a cut-off value of 53% amino acid identity used to differentiate rotavirus species [37,38,39]. Among the 11 different rotavirus species, RVA is the most common species that affects humans as well as other animals and, thus, is the focus of this review article. RVD, RVF, and RVG have only been identified in birds [40], while all others have been identified in humans and/or other mammals.

The two outer capsid glycoproteins VP7 and VP4, along with the intermediate capsid protein VP6, are the most immunogenic proteins of the virus and independently elicit neutralizing antibodies following infection [41,42,43,44,45]; VP7 is considered to have the major neutralizing epitopes of the virus [41]. Because of this and the dependent segregation of VP7 and VP4, they form the basis of a widely accepted binomial nomenclature by which RVA are further classified into G- and P-types based on the identity of the glycoprotein VP7 and protease-sensitive VP4, respectively [36,46]. The prevalence of different G-type and P-type strains vary geographically (both within and between geographical areas), as well as temporally. To date, at least 42 G-types and 58 P-types of RVA have been identified, and these tend to segregate according to species-specific patterns across the various susceptible animal species [2] (https://rega.kuleuven.be/cev/viralmetagenomics/virus-classification/rcwg, access on 26 December 2023). Thus, infections are typically limited to only a few combinations occurring in different animal species. In humans, infections with G1P[8], G2P[4], G3P[8], G4P[8], G9P[8], and G12P[8] strains of RVA are the most common [2,3]. Based on genogroup classification, these belong to either the Wa-like (genogroup 1, featuring a R1-C1-M1-A1-N1-T1-E1-H1 constellation and including predominantly G1P[8], G3P[8], G4P[8], G9P[8], and G12P[8] strains), DS-1-like (genogroup 2, featuring a I2-R2-C2-M2-A2-N2-T2-E2-H2 constellation and including G2P[4] and G12P[6], among other strains), or AU1-like gene segments (genogroup 3, featuring a I3-R3-C3-M3-A3-N3-T3-E3-H3 and including minor circulating strains of G3P[9] composition) (Table 2). These three genogroups are believed to have a close evolutionary relationship with porcine, bovine, and canine/feline RVA strains, respectively [46]. Comparatively, seven G-types (G3, G5, G6, G8, G10, G13, and G14) and six P-types (P[1], P[3], P[7], P[11], P[12], and P[18]) have been identified among equine RVA strains; however, the G3P[12] and G14P[12] genotypes are by far the most prevalent and epidemiologically relevant worldwide [12,47,48,49,50,51,52,53]. In recent years, the Rotavirus Classification Working Group (RCWG) has expanded this sequence-based nomenclature system to assign a specific genotype to each of the 11 genome segments according to established nucleotide percent cut-off values, allowing the classification of rotaviruses based on their overall genomic constellation [36]. Per this approach, RVA strains are designated based on VP7-VP4-VP6-VP1-VP2-VP3-NSP1-NSP2-NSP3-NSP4-NSP5/6 genes using the system Gx-P[x]-Ix-Rx-Cx-Mx-Ax-Nx-Tx-Ex-Hx (x = Arabic numbers starting from 1).

**Rotavirus entry and replication.** This review article is by no means a comprehensive analysis of the rotavirus replication cycle and will only focus on major or key steps related to the overall biology of this virus genus. The readers are encouraged to resort to other reviews or research articles on this topic for more detailed information [34,35,59,60,61,62,63,64]. Current knowledge of rotavirus replication is mostly based on well-characterized reference human and simian RVA strains; thus, replication strategies are assumed to be similar for equine and other animal rotaviruses.

Rotaviruses specifically infect mature enterocytes lining the apical portion of the small intestinal villi as well as enteroendocrine cells [65,66]. Virus attachment with subsequent penetration and uncoating are the initial series of steps preceding cytoplasmic replication [25]. The spikes along the outer capsid composed of VP4 serve as the main interacting viral proteins with host receptors [25]. The VP8* subunit of VP4 mediates attachment to sialic acids located on host receptors [67,68,69,70,71]. Additionally, sialoglycans such as GM1 and GD1a gangliosides serve as host attachment factors [72,73]. Neuraminidase treatment of cells can abolish infection of some strains, termed “sialic acid-sensitive” (e.g., simian RVA SA11), while some other strains are insensitive to neuraminidase treatment (e.g., human RVA Wa or DS-1) [74]. Histo-blood group antigens (HBGAs) have been recognized as additional virus-binding molecules on the host cell membrane, and such interactions have been shown to be dependent on the P-genotype [25,75,76]. Several integrins also serve as co-receptors and typically interact with specific motifs on the VP5* subunit of VP4 or on VP7 [74,77]. Following attachment, the VP4 undergoes conformational changes by adopting a “post-penetration umbrella” conformation; trypsin treatment has shown to favor this transition. The virus penetration process remains poorly understood, and it is believed that rotaviruses can enter via receptor-mediated endocytosis or direct membrane penetration with subsequent solubilization of the outer capsid and formation of DLPs [16,25,67]. Following cell penetration, DLPs become transcriptionally active, and the polymerase complex synthesizes all 11 species of positive-sense, single-stranded RNA, which are then released into the cytosol via the aqueous type I channels to be translated using the cellular machinery. They also accumulate within dense cytoplasmic inclusions known as viroplasms (Figure 2), viral factories formed by interaction between viral NSP2 and NSP5 [20]. Within viroplasms, NSP2 and NSP5 retain the +ssRNA molecules produced, recruit unassembled core proteins, and mediate viral genomic RNA packaging. The mechanisms driving assortment of the 11 +ssRNA segments in the viroplasm remain poorly understood. VP1, VP3, and +ssRNA interact to form the pre-core replication intermediates, subsequently followed by the addition of VP2 and formation of an active core replication intermediate in which dsRNA synthesis from +ssRNA takes place. VP6 is subsequently added to form DLPs, which translocate into the endoplasmic reticulum where they acquire the outer capsid composed of VP4 and VP7 via budding and acquisition of a temporary envelope. Mature particles are then released by cell lysis or non-classical vesicular transport to the plasma membrane [78].

**Pathogenesis.** Rotaviruses infect intestinal enterocytes and induce non-bloody diarrhea of typically short duration and elicit a limited inflammatory response in the small intestine [66]. These effects are primarily associated with the disruption of enterocytes lining the intestinal mucosa, with degeneration and loss of apical enterocytes leading to villous blunting/atrophy and fusion. Inflammatory infiltration of the affected intestinal mucosa is often mild (as for other enteric viral infections) and mainly features infiltrating mononuclear cells [65,79]. The molecular events and host–virus interactions at the enterocyte level have been primarily investigated using surrogate cell culture systems and animal models due to difficulties establishing human or other animal enterocyte-specific models though, currently, the use of intestinal organoids may shed light onto the enterocyte-specific events following rotavirus infection in animals and humans [80,81,82].

In suckling mice, diarrhea is typically limited to 5 days post infection (dpi), with viral shedding occurring up to 10 days post infection (dpi) [83]. The dynamics of viral infection are largely similar for other RVAs infecting other animal species such as calves, piglets, and foals [1,48,84,85,86,87,88,89,90]. Histologically, the enterocyte alterations are characterized by cytoplasmic vacuolation with an increase in apoptotic rate and downregulation of enterocyte-specific transcripts (e.g., lactase, liver fatty acid binding protein [L-FABP], and sodium glucose cotransporter 1 [SGLUT1]), which correlate with the villous atrophy observed) [83]. Villous blunting and fusion are common morphologic alterations noted across animal species [48,86]. Subsequently, an increase in cellular proliferation is noticeable at the level of the intestinal crypts, leading to repair of the epithelial surface. Hence, the combination of apoptosis, epithelial cell turnover with replacement by immature enterocytes, and enterocyte gene expression shutoff leads to the defective absorptive functions and causes the malabsorptive diarrhea that characterizes rotavirus infections [83].

Failure of ionic homeostasis featuring increased intracellular levels of Ca^2+^ has been implicated as at least one of the mechanisms of cytopathogenicity [91]. Studies performed in cell culture systems and mice have implicated the viral enterotoxin NSP4 as a virulence factor driving rotavirus pathogenicity [92,93]. RVA NSP4 mediates alterations in intracellular Ca^2+^ concentrations [94,95]. Interestingly, similar effects were identified with RVB NSP4, which suggests that the function of NSP4 might be conserved across rotavirus species/groups [96]. Furthermore, a domain within NSP4 (amino acids 47–90) has been recognized as a viroporin responsible for disruption of membrane integrity and ion homeostasis following oligomerization of NSP4 at the endoplasmic reticulum [94]. This domain contains a lysine cluster that directs membrane insertion, critical for viroporin activity. The use of NSP4 mutants also demonstrated that alterations in this domain prevent increases in cytosolic Ca^2+^ levels and the formation of viroplasm puncta [94]. The increase in intracellular Ca^2+^ leads to activation of Ca^2+^-dependent chloride channels, causing excess chloride egress into the intestinal lumen creating an osmotic gradient and leading to osmotic diarrhea [97].

In addition to the aforementioned roles of NSP4, this viral protein also induces other alterations, particularly to the cellular cytoskeleton [98]. Apical administration of NSP4 reduces transepithelial electrical resistance with redistribution of actin filaments and an increase in paracellular flux. Interestingly, exposure of pre-confluent cells to NSP4 prevents the development of a permeability barrier and the lateral targeting of the tight-junction-associated Zonula Occludens-1 (ZO-1) protein, thus impairing epithelial barrier recovery/repair by affecting tight-junction biogenesis [98]. Such effects could also be linked to the binding affinity of this non-structural viral protein for the basement membrane proteins laminin-β3 and fibronectin [95].

An additional component of rotavirus pathogenesis in the intestinal tract relates to its effect on the enteric nervous system, leading to its activation and subsequent increase in intestinal motility [65]. It has been demonstrated that the increase in intracellular Ca^2+^ mediated by NSP4 can trigger the secretion of 5-hydroxytryptamine (5-HT, serotonin) from enteroendocrine cells, inducing activation of enteric nerves and increasing intestinal motility as well as vagal nerve stimulation and induction of nausea and vomiting [99,100,101,102]. Thus, the mechanisms of rotaviral diarrhea are osmotic (due to malabsorption associated with direct enterocyte damage) and secretory (directly associated with the effect of the NSP4 virotoxin).

## 3. Human Rotavirus A: Epidemiology and Transmission

RVAs are ubiquitous in the human population and are a common infection in young children. Nearly every child becomes infected with RVA between the age of 3 and 5 globally [103]. While the prevalence of RVA infections is similar around the globe (~30–50%), the vast majority of the fatal RVA infections correspond to children living in low-income countries [104], likely associated with socioeconomic variables and limited access to health care. It has also been determined that, in low-income countries, these human RVA infections are typically associated with uncommon RVA strains (e.g., G9P[6]), occur at younger ages, and have a less pronounced seasonal distribution compared to high-income countries [104,105].

Implementation of RVA vaccination has reduced the burden of RVA disease, and among children <5 years of age the median percentage reductions in hospitalizations and emergency department visits due to RVA gastroenteritis were reduced by a median of 67% overall, and 71%, 59%, and 60% in countries with low, medium, and high child mortality, respectively, during the first decade following vaccine licensure [106]. Vaccination has also influenced demographics and the seasonal pattern of disease, with more frequent disease in unvaccinated children at older ages (6 to 16-year-old) and senior patients over 70 years old, as well as delayed and shortened the RVA season with blunting of disease peaks [107,108].

In humans, the most common genotypes are G1P[8], G2P[4], G3P[8], G4P[8], G9P[8], and G12P[8] [109] while others are infrequent but could pose an emergent pattern. Even though differences in circulating genotypes have been noted following vaccine introduction (e.g., the G2P[4] strains following implementation of the monovalent G1P[8] RVA vaccine), little evidence suggestive of selective pressure is available [110,111].

High titers of RVA are shed in the stools during episodes of associated diarrhea. Transmission occurs via the fecal–oral route, mainly via direct contact, though contaminated fomites in care settings and hospitals also play a significant role [112,113]. It has been estimated that as little as nine virions can induce disease in susceptible individuals [114].

## 4. Equine RVA: Epidemiology and Transmission

It has been well-established that equine RVA has a worldwide distribution, and serological analysis demonstrates that adult horses are frequently seropositive [115]. Thus far, seven G-types (G3, G5, G6, G8, G10, G13, and G14) and six P-types (P[1], P[3], P[7], P[11], P[12], and P[18]) have been identified in horses. The G3P[12] and G14P[12] genotype constellations are the most prevalent and circulate in horse populations around the globe [48,50,53,54,116]. The other G/P-type combinations reported to date (G3P[3] [50], G5P[7] [117,118], G8P[1] [119], G10P[11] [119,120], and G13P[18] [121]) are of rare occurrence. G3 strains are further classified into G3A and G3B based on five amino acid polymorphisms in three antigenic regions within VP7 identified via neutralization studies [122]. G3A rotaviruses have been frequently detected in Argentina [50], Australia [123], European countries [49,52,54,116,124,125], and the United States [48]. In contrast, only G3B rotaviruses circulated in Japan until the first identification of G3A rotaviruses in 2016 [51,53,126,127]. Importantly, in 2021, an emergent equine group B rotavirus (RVB) was identified as the etiological agent involved in localized outbreaks of diarrhea in Central Kentucky [128]. Equine RVB has been previously reported only once in a single study from Germany [129] and little is known about its distribution, prevalence, and pathogenicity compared to equine RVA. This further complicates diagnoses and vaccination strategies to prevent diarrhea in foals.

The occurrence of rotaviral diarrhea is usually seasonal, with most cases reported in winter, spring and early summer, and coincides with the foaling season [47,48]. Temporal fluctuations in the circulation of G3P[12] and G14P[12] strains have been recognized in molecular epidemiology studies carried out in Argentina and Japan; the reason for this cyclic pattern is not fully understood. Vaccination practices with vaccines that only include a G3P[12] strain have been speculated to play a role in the dynamics of circulating rotaviruses in those countries [51,53,130]. Molecular analysis of equine G3P[12] and G14P[12] strains have also shown a strong association with the VP6 genotypes I6 and I2, respectively, suggesting co-segregation of viral genome segment 6 and segment 9, which could have implications in rotavirus evolution [131] (Table 2).

Equine RVA affects young foals, typically less than 6 months of age. A recent study carried out by our group has determined that equine RVA-infected foals had an age range between 13 days and 4 months, with the highest frequency observed between 2 and 3 months of age (78.3% of the cases) [48]. While the morbidity tends to be high and outbreaks occur in farms every season, mortality is often low as long as appropriate supportive therapy is instituted [47]. Disease transmission occurs through the fecal–oral route, with virus shedding in feces that can reach up to 10^9^ virus particles per gram of feces [47]. This high environmental burden is also favored by the high resistance to inactivation that rotavirus particles have in the environment. Experimental infection studies performed in the late 1980s and early 1990s demonstrated that the incubation period in foals ranges between 1 and 4 days, with diarrhea ranging from 1 to 9 dpi and shedding starting at 1 dpi and extending to up to 12 dpi [12,132].

## 5. Impact of Rotavirus Infections on Human and Equine Health

Group A rotavirus infections have a significant impact on human as well as animal health. Nearly all animal species are susceptible to RVA-induced diarrhea during early stages of their life, causing life-threatening diarrhea. Even though vaccination has significantly reduced its incidence, rotaviruses are still considered to be the leading cause of severe dehydrating diarrhea in children aged less than 5 years globally. A 2003 study estimated the global illness and deaths caused by rotavirus disease by reviewing data published between 1986 and 2000, before the recommendation by the World Health Organization (WHO) to implement rotavirus vaccination in national vaccination programs in 2006 [103]. That study showed that this viral infection causes overall 138 million cases of gastroenteritis each year, of which 111 million required only home care, 25 million required clinic visits, and 2 million needed to be hospitalized, with 352,000–592,000 deaths in children less than 5 years of age. Based on that analysis, by the age of 5, every child would have had an episode of RVA diarrhea with 1 in 5 visiting a clinic, 1 in 60 being hospitalized, and 1 death per 293 cases. Geographically, 82% of deaths due to RVA diarrhea are among children in countries of low socioeconomic status.

In 2006, the WHO first recommended the inclusion of rotavirus vaccination in national immunization programs in the European region and America. It was not until 2009 that the WHO recommended this practice be extended to all regions worldwide [133,134,135]. Such vaccine programs have been successful in reducing the burden of rotaviral disease worldwide, and disease outbreaks have, therefore, often been linked to vaccination interruptions [136]. In the US, the vaccination program led to a 53–93% reduction in rotavirus positivity compared with the median in 2000–2006 [108,137,138]. A more recent study evaluating global burden between 1990 and 2019, based on the Global Burden of Disease 2019 Study, concurred that rotavirus infections were the leading cause of deaths associated with diarrhea globally, accounting for 19.11% of diarrhea-associated deaths in 2019 [10]. That study also determined that the age-standardized death rate (ASDR) of rotavirus declined from 11.39 per 100,000 people in 1990 to 3.41 per 100,000 people in 2019, with an average annual percentage change of −4.07%. Countries with a low sociodemographic index (SDI) had the heaviest burden of rotavirus infections with an ASDR of 10.71 per 100,000 people, specifically in African, Oceanian, and South Asian countries. Interestingly, there was an uptrend in the ASDR in the high–middle SDI region between 2017 and 2019 (annual percentage change of 5.87%). In summary, even though implementation of vaccines has significantly reduced the incidence of rotavirus gastroenteritis globally, the burden of rotavirus infections in the human population remains high, particularly in low-income countries; therefore, continued implementation of effective public health policies, increased coverage using existing vaccines, and development of more effective vaccines is needed to counteract this burden.

Comparatively, global prevalence studies to assess equine RVA burden in the horse population are lacking, and prevalence studies are limited to defined geographical regions. Such studies have established that rotavirus infections have the highest impact as a cause of diarrhea in foals, being responsible for 21% to up to 34.6% of foal diarrhea cases [48,50,139]. No studies have been undertaken to comprehensively determine the specific economic impact of rotavirus infections in foals specifically; however, according to an informal study by the Ontario Veterinary College, there are three to four fatalities per every ten horses with acute diarrhea [140]. There are several commercial vaccines to prevent equine RVA diarrhea currently available. The mares are usually immunized with three doses at 8, 9, and 10 months of gestation to elicit RVA-specific antibody titers in serum and concentrated in the colostrum. Antibodies are passively transferred to their foals via colostrum intake and aid to delay the onset of RVA infection and diarrhea, reducing viral shedding, severity, and duration of diarrhea. However, its efficacy in preventing infection is low and we have demonstrated that 82.6% of infected foals were born from vaccinated mares [48]. One potential reason for this might be associated with the lack of G14P[12] strains in vaccine formulations and the overall prevalent circulation of G14P[12] RVA strains [141] (Papp review), as there is little to no cross protection among these genotypes.

Hence, equine RVA infections continue to be a significant health burden to breeding enterprises all over the world. Comprehensive surveillance and coordination between veterinary diagnostic laboratories and clinics around the globe would be required in order to assess global trends of equine RVA infections. More efficacious and comprehensive vaccines (i.e., including G14P[12] genotype strain(s)) are also needed to diminish the negative impact of rotavirus infections on breeding farms.

## 6. Viral Evolution and Emergence of Rotaviruses That Cross Species Barriers

RVA are a genetically diverse group of segmented RNA viruses in which multiple sources of genetic variation have been identified. In 2008, the Rotavirus Classification Working Group (RCWG) proposed and implemented a new classification system for RVA based on whole genome sequencing [36,142]. Under this system, RVA are classified based on the 11 genome segments, assigning specific genotypes based on nucleotide homology and set cut-off parameters. To designate the full genetic make-up, the nomenclature Gx-P[x]-Ix-Rx-Cx-Mx-Ax-Nx-Tx-Ex-Hx representing each genome segment (VP7-VP4-VP6-VP1-VP2-VP3-NSP1-NSP2-NSP3-NSP4-NSP5 genes) is used, where the Arabic number “x” represents a genotype number (e.g., G1, P[4], I2, etc.). This system combined with whole-genome sequencing has enhanced our understanding of different aspects associated with rotavirus evolution and molecular epidemiology, including determination of origin, assessment of genetic diversity, identification of reassortment, and antigenic drift, among other mechanisms driving evolution, interspecies transmission, and emergence of novel strains.

Rotavirus evolution is dependent on the interaction between variation in viral genomic composition, host adaptation, and evasion of host immunity [17,143]. The mechanisms driving genomic variation include point mutations (antigenic drift), reassortment (antigenic shift), rearrangement, and recombination, or (most commonly) a combination of all aforementioned processes [2,14,17,136,143]. It is speculated that point mutations and reassortment constitute the main drivers of rotavirus evolution. As for other RNA viruses, point mutations occur spontaneously due to the error-prone activity of the viral RdRp (VP1); this could be as high as 5 × 10^−5^ per nucleotide [144], roughly indicating that at least one mutation is likely to arise for every new genome copy synthesized. While most of these represent synonymous changes, the majority of the non-synonymous changes seem to occur within key neutralizing epitopes on surface proteins, leading to immune selection and circulation of strains that have an impact on vaccine efficacy [143]. In line with this, it has also been demonstrated that the antigenic epitopes on VP7 and VP4 assigned to the same G- and P-type may still frequently exhibit amino acid variation and, consequently, affect the efficacy of neutralizing antibodies; this, in turn, can affect vaccine efficacy, even against viruses belonging to the same genotype. Reassortment is an important mechanism of viral diversity for segmented RNA viruses by which two or more closely related viruses exchange specific genome segments during co-infection and replication within a single host cell, leading to emergence of new genotype constellations [145,146]. A critical factor for the success of this process is the frequency of co-infection. This phenomenon has often been involved in the zoonotic transmission of rotaviruses (see below). Based on previous studies, it has been estimated that the rate of co-infection can be as high as 20% in developing countries, while in developed countries this rate is <5% [136]. This may explain why the genetic diversity of rotaviruses circulating in developing countries is higher compared to developed counterparts. Also, the attenuated strains used for vaccination can donate genome segments to the circulating pool of human viruses via reassortment; the identification of NSP2 genes derived from the vaccine strain used in the RotaTeq^®^ vaccine in rotaviruses shed by sick children in Nicaragua serves as an example [147].

Other mechanisms of rotavirus evolution involve rearrangement (or intragenic recombination) and recombination (both intratypic and intertypic). Rearrangement includes the occurrence of insertions, deletions, and duplications within coding regions. This most commonly occurs as duplications on the 3′ end of rotavirus genome segments; however, its evolutionary benefits are unclear. Conversely, true recombination requires co-infection of a single cell by two or more rotavirus strains with consequent recombination of gene fragments between strains [143]. This has been reported for the genome segment encoding the outer capsid protein VP7, but more recent studies described this as a rare phenomenon not leading to subsequent transmission.

**Figure 3:**
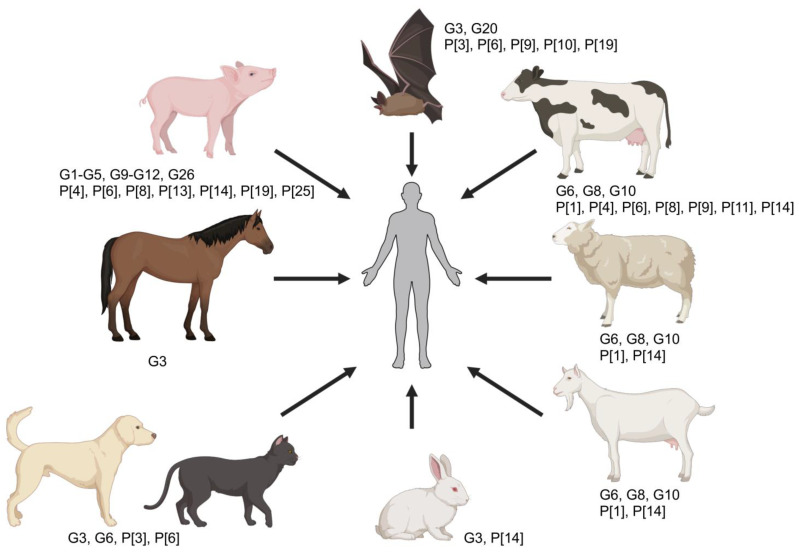
Zoonotic transmission of group A rotaviruses from animals to humans. Animal rotaviruses significantly contribute to the gene segment pool of human RVA strains, enhancing their genetic diversity. Specific G- and P-types transmitted from animals to humans are depicted for each animal species.

While initially believed that RVA species had a host-restricted tropism, there is increasing evidence—especially via sequencing and phylogenetic analysis of genomic segments—that transmission to other animals or humans (interspecies transmission) is a common event, and that animal rotaviruses significantly contribute to the genetic diversity of human RVA strains (Table 2 and Figure 3) [2,14,17,143,148,149]. Interspecies transmission of rotaviruses can occur either via direct transmission to a new host or subsequent to genetic reassortment between heterologous strains [143]. Since zoonotic transmission (i.e., infection of humans by a rotavirus strain recognized to infect animals) has been only sporadically identified [109,150], it has, therefore, been concluded that direct transmission events (from animals to humans or vice versa) are rare. Examples of such includes the human RVA strains G3P[3] Ro1845 and HCR3A, which share all genomic segments with canine (CU-1, K9, A79-10) and feline (Cat97) strains [151], as well as the human B4106 G3P[14] strain with the lapine strain 30/96 G3P[14] (Table 2) [152,153,154]. However, the low transmissibility rate in the new host usually results in a dead-end infection following direct transmission, and this is likely determined by both host and viral genetic factors (e.g., NSP1).

In contrast, viral reassortment is a common evolutionary mechanism leading to more efficient interspecies and zoonotic transmission, and human rotavirus strains of animal origin are constantly being reported across the globe. Such events are commonly reported in many developing countries (where co-infection rates are high; see above), and the high rate of co-infection and genetic diversity likely explain the reduced vaccine efficacy and higher rates of infection in these areas of the world. Reassortant strains typically carry one or more animal-origin gene segments amidst a genetic background of human-derived RVA strains that favor a higher transmissibility rate and have been isolated from infected children in both developed and developing countries; hence, the human–animal interface plays a significant epidemiological role and livestock, and other animals are regarded as reservoirs for human infection. While G3 (derived from cats, dogs, non-human primates, pigs, mice, rabbits, and horses), G5 (derived from pigs); G6, G8, and G10 (derived from cattle and other ruminants); and G9 (derived from pigs) strains have been detected in the human population [2,153,154,155,156,157,158,159], it is the porcine and bovine RVA strains that mostly contribute to the emergence of viral reassortants in the human population (Table 2 and Figure 3). Recently, horses have been implicated as the source of a novel equine-like G3 RVA infecting children around the world [15,55,160,161]. The contributions of each of these animal species to the emergence of animal-like rotavirus reassortants in the human population are discussed next; however, it is important to clarify that these are not the only animal rotaviruses that contribute to human rotavirus diversity and the list is more comprehensive yet beyond the scope of this review article.

**Porcine-derived human rotaviruses.** A vast number of RVA strains of porcine origin have been identified in human patients, thus there is evidence that porcine-derived human RVA strains have occurred and spread more successfully in the human population compared to other animal-derived rotaviruses [2,11,162]. In a recent review article, a total of 10 G genotypes (G1-5, G9-12, and G26) and 7 P genotypes (P[4], P[6], P[8], P[13], P[14], P[19], and P[25]) have been identified as porcine in origin with either regional occurrences, a more widespread occurrence around the globe, or with emerging features [11]. In line with this, the most recent full-genome-based classification of rotaviruses has demonstrated that the most widespread human rotavirus strains corresponding to the Wa-like genogroup/genogroup 1 (i.e., G1P[8], G3P[8], G4P[8], and G9P[8]) share the majority of the genomic segments with porcine rotavirus strains (R1-C1-M1-A1-N1-T1-E1-H1; corresponding to VP1, VP2, VP3, and NSP1 through NSP5), suggesting a common origin between porcine and human Wa-like rotaviruses (Table 2) [46]. Interestingly, two of the G genotypes that have emerged globally in recent years (i.e., G9 and G12), and which currently circulate in all continents, are also believed to be of porcine origin [2]. Another example is that of G5 strains, the most common G-type circulating in piglets, which have been detected in children in various South American countries [163]. Other more infrequent strains include G11P[25] strains, such as one detected in South Korea with a G11-P[25]-I12-R1-C1-M1-A1-N1-T1-E1-H1 genotype constellation determined to be a human–porcine reassortant of two distant relatives of the G11 strains circulating in the world [164]. It is hypothesized that the interactions between VP4 and HBGAs and sialic acids could be associated with the regional prevalence and zoonotic potential of some of these porcine-derived viruses. Hence, regional distribution of polymorphisms in the HBGAs across the human population could explain regional differences in the occurrence of certain RVA strains derived from pigs [11,165].

**Bovine-derived human rotaviruses.** Bovine RVA strains have also significantly contributed to the genetic diversity and emergence of novel human strains in multiple instances. Bovine-like VP7 (G6, G8, and G10) and VP4 (P[1], P[2], P[4], P[6], P[8], P[9], P[11], and P[14]) were detected around the world (Table 2 and Figure 3) [2,46,166]. Genome constellation analysis has shown that, similarly to how Wa-like human strains shared genomic segments with porcine rotaviruses, the human DS-1-like genogroup/genogroup 2 (mainly characterized by G2 and P[4]) shares a large proportion of genomic segments with bovine rotavirus strains, mainly I2-R2-C2-M2-N2-E2 (corresponding to VP6, VP1, VP2, VP3, NSP2 and NSP4) (Table 2). While a lower proportion of gene segments in common have been identified, these findings strongly suggest a common origin between DS-1-like and bovine rotavirus strains [46]. A larger analysis of human G2P[4] strains in the post-vaccination period in Belgium demonstrated that these were very closely related to bovine-like rotaviruses and at least eight bovine-like–human interspecies reassortment events on seven different gene segments (VP6, VP1, VP2, VP3, NSP2, NSP4, and NSP5) were identified. Such reassortment events likely led to the successful spread of this specific genotype as the Rotarix^®^ vaccine implemented in Belgium had a lower efficacy against DS-1-like compared to Wa-like rotaviruses [166]. While it remains unclear whether reassortment between human and bovine rotaviruses is restricted to certain gene segments, it seems that gene segments sharing the same genotype in human and ruminant rotaviruses (VP1-VP3, VP6, NSP2, and NSP4) may reassort without significant loss in fitness [166]. Interestingly, an emergence of human rotaviruses with the bovine-like NSP4 gene has only been observed in several countries around the world [166].

The bovine-like strain G10P[11] has been detected regionally in the Indian subcontinent since 1993 and contains nine genomic segments derived from bovine strains [167,168]. Similarly, two G8P[14] and one G10P[14] human rotaviruses identified in Italy carried a nearly complete bovine-like genome constellation (I2-R2-C2-M2-A3/A11-N2-T6-E2-H3), with genetic distances suggesting two interspecies transmission events to humans [169]. In Sub-Saharan Africa, some African rotavirus strains (specifically, G8P[6] and G8P[8]) derived from a reassortment event between a human G2P[4], a human DS-1-like, and strains carrying G8, P[6], or P[8] genotypes have been demonstrated [170]. Many more instances of interspecies transmission have been detected than can possibly be covered in this article, which highlights the plasticity of RVA strains and the need for continued monitoring at the human–animal interface.

**Emergence of an equine-like G3 human rotavirus A.** G3 strains infecting humans and a wide range of animal species are among the most genetically diverse rotaviruses, with at least nine lineages identified [56]. In humans, G3P[8] strains are the most commonly reported G3 strains [171] and primarily belong to G3 lineage I and a Wa-like genogroup 1 constellation (G3-P[8]-I1-R1-C1-M1-A1-N1-T1-E1-H1) [56,171]. In recent years, novel human rotaviruses representing potential reassortment events between human and equine rotaviruses have been detected and spread globally. Specifically, since 2013, a novel DS-1-like intergenogroup reassortant with a VP7 of equine origin and designated as “equine-like G3” has emerged in the human population and rapidly spread to many countries across five continents, in some instances with endemic circulation [15,55,57,58,171,172,173,174,175,176,177]. The first report dates to April 2013, when two novel G3P[4] rotavirus strains were detected in children suffering from acute gastroenteritis in Sendai, Japan [15]. Compared to common human G3 (lineage I) strains with a Wa-like genogroup 1 genomic constellation, these novel strains have a lineage IX G3 [56] and a DS-1-like genogroup 2 constellation of G3–P[4]–I2–R2–C2–M2–A2–N2–T2–E2–H2 (designated RVA/JPN/S13-30/2013/G3P[4] and RVA/JPN/S13-45/2013/G3P[4], respectively [Table 2]) [15]. Specific analysis of the genome segment 9 (VP7) of these strains clearly demonstrated a high nucleotide identity and close phylogenetic relationship with an ERVA G3 strain identified in India (RVA/Horse-wt/IND/Erv105/2004-05/G3P[X]; DQ981479.1), constituting the first report of a putative genetic reassortment between human and equine rotavirus and regarded as the prototype equine-like G3 strains. While no additional equine-like G3 strains possessing a P[4] have since been reported, temporally concurrent with the detection of the above novel human–equine reassortants, the Australian Rotavirus Surveillance Program reported the emergence of G3P[8] strains as the second most common genotype across Australia in the 2013 annual report. These G3P[8] strains represented 31% of all specimens examined in the reporting period and possessed a G3-P[8]-I2-R2-C2-M2-A2-N2-T2-E2-H2 genome constellation, which is considered a double (VP7 and VP4) intergenogroup reassortant between an unknown novel G3 strain (perhaps the G3P[4] identified in Japan) and a DS-1-like G1P[8] strain (Table 2) [55,161]. This genotype was the most commonly identified among children less than 5 years old (39.2% of samples) and the dominant genotype in the Northern Territory and Western Australia, comprising 93% and 44% of the positive samples, respectively. Preliminary sequencing data demonstrated an unusual G3 VP7 protein more closely related to equine G3 strains than other human strains, which could explain its emergence. The Australian Rotavirus Surveillance Program performed further genotypic analysis from a total of 518 rotavirus-positive fecal samples derived from children aged less than 5 years, among which 193 samples corresponded to a G-non-typable P[8] RVA strain. Sequencing of the VP7 gene demonstrated highest nucleotide identity with the human-equine reassortant strains identified earlier in Japan (RVA/JPN/S13-30/2013/G3P[4] and RVA/JPN/S13-45/2013/G3P[4]) and the equine strain RVA/Horse-wt/IND/Erv105/2004-05/G3P[X]. This emergent strain was divergent to the majority of human strains and was classified as an equine-like G3P[8] strain. Retrospective analysis demonstrated that it was first identified in April 2013 and circulated until December 2014 in Australia. Antigenic analysis showed that the equine-like G3P[8] strains had a distinct antigenic profile compared to previously circulating G3P[8] strains [161].

Since its first detection in Japan and Australia in 2013, this rotavirus strain has been reported in at least 14 countries across five continents (Asia, Australia, Europe, North America, and South America) [55,57,58,161,172,174,175,177], with a significant prevalence in many countries, including Indonesia, Spain, Germany, Brazil, and the Dominican Republic, among others. In 2015, this strain was detected in children in the US [174]. Strikingly, in some locations, these strains have become highly prevalent in the human population. For example, a study performed between 2017–2019 in Sicily, Italy has demonstrated an increase from 7% to 71% of equine-like G3P[8]-positive cases within this 2-year period [178]. This has resulted in a globally circulating pool of highly conserved “equine-like” G3 strains, with a distinct genetic constellation and with potential to further reassort with other strains belonging to both genogroups 1 and 2 [56]. Such potential to further reassort has been highlighted by the emergence of additional reassortant strains comprising genome constellations G3-P[8]-I2-R2-C2-M2-A2-N1-T2-E2-H2 (triple VP7, VP4, and NSP2 reassortant; [58,175]) or G3-P[6]-I2-R2-C2-M2-A2-N2-T2-E2-H2 [57] (Table 2).

A study performed after the first identification of this novel strain in Japan (2016–2017) demonstrated that no clinical differences exist between equine-like and other G3 strains [160]. Overall, based on genome constellation analysis, it was hypothesized that these strains originated from Southeast Asia. Importantly, the VP7 gene of the genotyped strains during that time clustered distantly to other human G3 strains (with only 81–82% nucleotide identity) but more closely to animal G3 strains (nucleotide identity ranging between 81–91%), with the equine Erv105 G3P[X] once again the closest strain with roughly 91% nucleotide similarity. While this could indicate equine origin, the fact that the VP7 gene of the Erv105 strain reportedly has a low nucleotide identity to other equine RVA strains (approximately 87%) could also suggest an alternative origin in either another unknown animal source or, as proposed by some authors, from a human G3 strain that initially infected an equine host [56]. Continued surveillance/genotypification is, therefore, warranted to elucidate the origin of these emerging reassortant equine-like G3 strains. This information is also critical for the rationale design of vaccine candidate strains.

The study by Bonura et al. [178] has also shown that the equine-like G3P[8] circulating in Italy is characterized by multiple amino acid substitutions in neutralizing epitopes of VP7 compared to the Rotateq^®^ and Rotarix^®^ vaccine strains, while conserved compared to other global equine-like G3 strains (T87S, N213T, K238D, and D242A). The impact of these substitutions on vaccine efficacy remains to be fully addressed.

**Figure 4:**
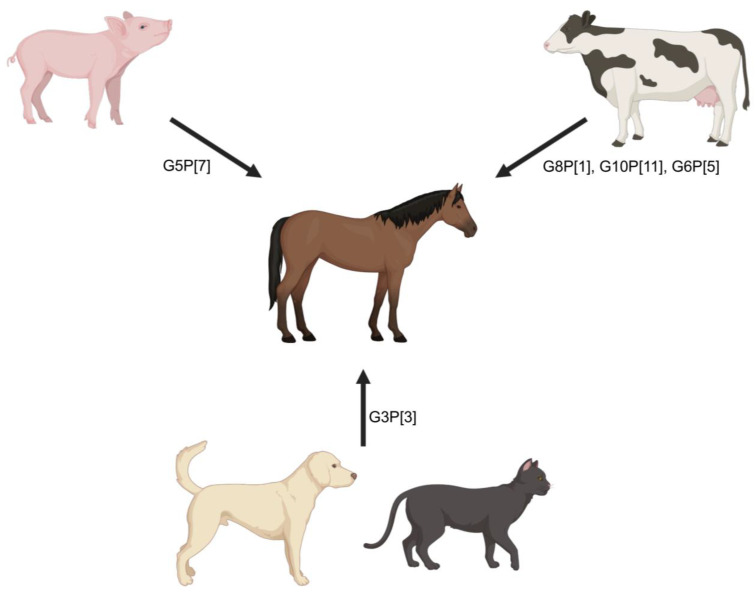
Cross-species transmission of infrequent RVA G- and P-types from other animals to horses.

**Cross-species transmission to horses.** Unfortunately, comprehensive surveillance studies of equine RVAs are limited both geographically and by the amount of whole genome-level data available for strains previously and currently circulating in the horse population. Geographically, most surveillance studies are regionally limited, with no global-level studies that would allow thorough assessment of transmission events, reassortments, or future transmission risks. Based on the available viral genomic data, cross-species rotavirus transmission from other animals to horses has been reported in multiple instances. Among the infrequent genotypes identified in horses, G8P[1], G10P[11], G3P[3], G5P[7], and G6P[5] equine RVAs are regarded to be of other-animal origin including bovine/bovine-like human rotaviruses (G8P[1], G10P[11], and G6P[5] [119,179]), feline and canine rotaviruses (G3P[3]), or porcine rotaviruses (G5P[7] [117,180]) (Figure 4). However, none of these genotypes are epidemiologically relevant and do not actively circulate in the horse population, which is an indication of limited host adaptation.

## 7. Rotavirus Vaccines

***Vaccines for human use.*** Based on the facts that RVA infections occur predominantly during the first year of life, that reinfections after 2–3 years of age are not associated with gastroenteritis, and that protection following natural infections is associated with the presence of antibodies in serum and within the intestinal lumen (in the form of rotavirus-specific IgA) [181,182,183], rotavirus infections were recognized as likely to be vaccine-preventable [184]. The first live-attenuated quadrivalent vaccine that was licensed following phase III trials was the RotaShield^®^ (Wyeth Lederle Vaccines, Pearl River, NY, USA) in 1998 [185,186,187,188]. This vaccine was composed of the attenuated simian RVA RRV strain (G3P7[5]) and mono-reassortants from this virus containing human RVA G1, G2, and G4 VP7 genes. The vaccine was rapidly applied as a universal vaccine following licensure to over 1 million children in the US, but the occurrence of intestinal intussusception in vaccinees led to voluntary discontinuation by the manufacturer.

Two vaccines (namely Rotarix^®^ (GlaxoSmithKline, Brentford, UK) and RotaTeq^®^ (Merck, Rahway, NJ, USA)) have been licensed and became available for the prevention of RVA infections in children in several countries around the world in 2005 and 2006, respectively [189,190]. In 2006, the WHO issued a recommendation to incorporate such vaccines in national immunization programs in the European region and America [135]. The US Advisory Committee on Immunization Practices subsequently recommended Rotateq^®^ (2006) and Rotarix^®^ (2008) for universal vaccination of infants [191,192]. Thereafter, the WHO extended the recommendation for inclusion of rotavirus vaccination in all national immunization programs globally in 2009 [134]. These vaccines have resulted in significant reduction in rotavirus-associated healthcare, with most success observed in developed countries around the world (and references therein).

Each of these licensed vaccines are live-attenuated and administered orally to infants at 2 and 4 months of age (Rotarix^®^) or 2, 4, and 6 months of age (RotaTeq^®^), respectively [133,192]. The Rotarix^®^ vaccine is monovalent and derived from the human RVA G1P[8] RIX4414 strain, isolated from an infected child (isolate 89-12) and attenuated following serial cell culture passage [189,193]. Based on its genotype, this vaccine elicits protective responses to most of the globally circulating human RVA strains (G1P[8], (G1P[8], G2P[4], G3P[8], G4P[8], and G9[P8]); induction of sufficient heterotypic responses against G2P[4] strains resulting in sufficient levels of protection has been reported, although the specific immune mechanisms underlying this heterotypic response remain unclear.

In contrast, the RotaTeq^®^ vaccine is a pentavalent vaccine composed of five human-bovine reasssortant viruses (WI79-9 (G1P[5]), SC-2 (G2P[5]), WI78-8 (G3P[5]), BrB-9 (G4P[5]), and WI79-4 (G6P[8])) [190,194]. These reassortants were generated via reassortment between the bovine RVA strain WC3 (G6P7[5]) with each of the five human RVA strains, the latter of which contributed with a G1, G2, G3, or G4 VP7 or P[8] VP4 gene [195]. This multivalent design approach incorporates VP7 (G) or VP4 (P) genes from the globally dominant human RVA genotypes actively circulating (listed above) and increases vaccine efficacy in the field as a protective responses to RVAs are mostly homotypic in nature, with demonstrated limited heterotypic protection [136,196].

Interestingly, while vaccination effectiveness has been high in developed countries (70 to 90%) [17], its efficacy has been estimated to be 20–30% lower in middle- and low-income countries. The underlying reasons for such differences are not currently understood and could be associated with nutritional and other factors [17,136,197,198,199,200,201].

Among the disadvantages of current live-attenuated vaccines, the limited heterotypic protection against other human RVA strains beyond those included in the vaccine formulation and also the occurrence of rotavirus reassortants between strains included in the vaccine formulation and wild-type rotaviruses are regarded as the most significant. Thus, several experimental vaccines are currently under development as alternatives. Among these are inactivated, subunit, norovirus nanoparticles expressing rotavirus VP8* proteins, virus-like particles (VLP), and plant-based VLPs expressing several rotavirus capsid proteins and mRNA vaccines [202,203,204,205,206,207,208,209,210,211].

***Vaccines for animal use:*** Vaccines are commercially available for the prevention of rotavirus infections in cattle, swine, and horses, species in which rotaviral diarrhea can cause devastating economic effects. Most of the vaccines commercially available are inactivated, with a few being live-attenuated, and are typically applied to pregnant animals in order to elicit strong maternal (passive) immunity that will provide early protection against neonatal diarrhea to newborn animals [84,212].

***(1) Vaccines against bovine RVA****:* Bovine RVA is a common agent associated with neonatal diarrhea in calves. Among the G- and P-types, only G6, G8, and G10 combined with P[1], P[5], and P[11] are considered epidemiologically important [213,214]. The G6 genotype accounts for roughly 40 to 78% of circulating bovine RVA strains, followed by G10 (21%) and G8 (3%, mostly in Africa). P[5] strains are the most prevalent (37 to 50%) followed by P[11] (15 to 35%) and P[1] (2%) [215]. The G6P[5], G6P[11], and G10P[11] combinations are the most predominant [148,213,214]. Both live-attenuated and inactivated vaccines against bovine RVA are commercially available for the vaccination of cattle, typically as multiple antigen vaccines combined with other micro-organisms associated with calf diarrhea [212]. Most of these share an antigenic composition (i.e., they include the same bovine RVA strains), typically bovine RVA G6 and G10 genotype strains. The inactivated vaccines are the most common and some of the commercially available vaccines include ScourGuard^®^ 4K and ScourGuard^®^ 4KC (Zoetis Animal Health), Fencovis^®^ (Boehringer Ingelheim), Bovilis^®^ Rotavec^®^, and Bovilis^®^ Guardian^®^ (Merck). ScourGuard^®^ 4K includes inactivated preparations of bovine RVA (G6 and G10), bovine coronavirus, and enterotoxigenic strains of *E. coli* containing K99 pili adherence factor, and ScourGuard^®^ 4KC additionally includes bacterin-toxoid from *Clostridium perfringens* type C. The Fencovis^®^ vaccine has a similar antigenic composition to ScourGuard^®^ 4K. The Bovilis^®^ Rotavec^®^ and Guardian^®^ both contain two bovine RVA G6 strains, namely NCDV-Lincoln (G6P[1]) and UK-Compton (G6P[5]), among other antigens to other agents. These inactivated vaccines are recommended for parenteral administration to pregnant cows during late gestation to elicit a strong maternal immunity and confer (partial) protection to the newborn calves [212,216,217,218,219,220,221,222]. Vaccine failure or breakthroughs have been reported and, among management factors in play, differing antigenic properties between vaccine and field strains have been responsible for low efficacy in these situations [212,223].

There is only one live-attenuated formulation from Zoetis Animal Health (Calf-Guard^®^), which is composed of attenuated strains of both bovine RVA and bovine coronavirus. This vaccine is not only recommended for vaccination of pregnant cows, rather for oral vaccination of newborn calves promptly following birth.

Very few alternative vaccines against bovine RVA have been developed and evaluated experimentally, and these are limited to rotavirus-like particles, *E. coli*-based VP8* subunit, baculovirus-based VP6 subunit, and DNA (VP4) vaccine candidates [224,225,226,227]. Overall, vaccination of pregnant cows demonstrated at least partial field efficacy, with a reduction in the duration and severity of calf diarrhea and virus shedding in feces. Future improvements in vaccine efficacy requires, at the very least, the incorporation of more contemporary strains in addition to the use of modern vaccine design. As an alternative to vaccines, passive immunity-based therapies utilizing anti-RVA IgY (IgY DNT) that are administered orally have been evaluated in dairy farms for the control of bovine RVA during the first 2 weeks of life. These have proven to be effective to complement current preventative vaccination practices [228].

***(2) Vaccines against porcine RVA***: Porcine RVAs are also a cause of weaning and postweaning enteritis in piglets. Prevalence varies from 3.3% to 67.3%, often with a higher farm-level prevalence despite available vaccines [11]. The most prevalent G- and P-types in swine include G5 (71.43%), G4 (8.19%), G3 (3.57%), G9 (2.31%), G11 (1.89%), G10 (1.26%), G1 (1.05%), P[7] (77.22%), and P[6] (12.07%) [11,214,229]. Historically, the most common genotype combination was G5P[7], but recent analysis of US porcine RVA strains between 2004–2012 identified G9P[13] as the most dominant (detected in 60.9% of positive samples (from Ohio (OH), North Carolina (NC), and Michigan (MI)]), followed by G9P[7] (8.7%), G4P[13] (8.7%), G11P[13] (4.3%), and G11P[7] (4.3%), while no G5 strains were detected [230]. Such findings are likely related to spatio-temporal fluctuations and genotype re-emergence [11].

Currently, there are two vaccines licensed by the United States Department of Agriculture (USDA) for use in the prevention of porcine RVA, namely ProSystem^®^ Rota and ProSystem^®^ RCE (Merck Animal Health, Madison, NJ, USA) [11,212,231]. ProSystem^®^ Rota is a live-attenuated vaccine containing the attenuated porcine RVA strains G5P[7] OSU, G4P[6] Gottfried, and G7P[7] A2. Alternatively, ProSystem^®^ RCE is multivalent and includes the strains indicated above in addition to antigenic preparations for other microorganisms including *Clostridium perfringens* type C and various *E. coli* pilus antigens. ProSystem^®^ RCE is administered to pregnant sows to boost maternal immunity while ProSystem^®^ Rota can be administered orally (first dose) and intramuscularly (second dose) to piglets seven to ten days before weaning. The latter vaccine is also commonly given to gilts to acclimate them prior to entering the sow farm as well. Field testing and usage data for both vaccines are scarce and, hence, a true estimate of their efficacy cannot be established; however, as for other animal rotaviruses, total protection is not achieved and infections continue to affect swine producers. Additionally, the contribution of live-attenuated vaccines to the genetic diversity of porcine RVA, leading to the emergence of novel variants, has been suggested for human RVA vaccines. Thus, alternatives to generate safer and more efficacious vaccines than current live-attenuated vaccines are critically needed. Experimental vaccines against porcine RVA have not been widely developed and tested and have been limited to subunit and virus-like particle approaches with limited success [232,233,234,235]. More recently, vaccine manufacturers such as Merck Animal Health (Rahway, NJ, USA) and Harrisvaccines (Ames, IA, USA) have utilized an RNA particle technology for the development of either prescription vaccines based on farm-specific VP7 sequences from circulating RVA strains (Sequivity^®^) or a porcine rotavirus group C (RVC) vaccine (using SirraVax^SM^ RNA Particle Technology, Harrisvaccines) to circumvent challenges in isolating and propagating RVC for conventional vaccine design. Improvement of current vaccine platforms for inducing protective responses against porcine RVA is still critically needed.

***(3) Vaccines against equine RVA:*** Genotypes of equine RVA have been described in detail in previous sections. Inactivated vaccines against equine RVA are available in several countries for the prevention of rotaviral diarrhea in foals. Three types of inactivated vaccines are available in different countries with variations in their composition. An inactivated vaccine based on the cell-culture-adapted H2 (G3P[12]) strain of equine RVA (RVA/Horse-tc/GBR/H-2/1976/G3P[12]) is licensed by Zoetis Animal Health (Kalamazoo, MI, USA) in various countries including the United States, New Zealand, Australia, and European countries (e.g., United Kingdom and Ireland). A variation of this inactivated vaccine is manufactured and has been available in Argentina since 1996 (Rotamix Equin, Biochemiq (Buenos Aires, Argentina)), which includes the H2 strain of equine RVA as well as the bovine RVA NCDV-Lincoln G6P[1] strain (RVA/Cow-tc/USA/NCDV-Lincoln/1967/G6P[1]), the simian RVA SA11 G3P[2] strain (RVA/Simian-tc/ZAF/SA11/1958/G3P[2]), and the *E. coli* antigen [47,50,84,141,236]. A different inactivated vaccine formulated with the equine RVA strain HO-5 (G3BP[12]) strain (RVA/Horse-tc/JPN/HO-5/1982/G3P[12]) was licensed in Japan in 2001 (Nisseiken, Tokyo, Japan; [237]). These inactivated vaccines are applied intramuscularly to pregnant mares typically at 8, 9, and 10 months of gestation to ensure appropriate colostral immunity to the foal and subsequently prevent rotaviral diarrhea [47,84,236,238,239]; the vaccination of foals is considered ineffective, at least with current inactivated vaccination approaches, as rotavirus infections occur very early in life, before waning of colostral immunity. These vaccines have been shown to elicit neutralizing antibody responses in the mare that are effectively transferred to the foal, and aid in reducing the incidence and severity of diarrhea and also in the titer and duration of viral shedding; however, they do not guarantee full protection and foals can still become infected [47,84,236]. The source of limited vaccine efficacy is likely multifactorial and may include the type of vaccine used (inactivated), the method of immunization and adjuvant(s) used in the formulation, the limited effectiveness of neutralizing antibodies to localize within the intestinal lumen of the foal, and the limited strain composition (only G3P[12] due to difficulties in obtaining cell culture-adapted field strains of equine RVA) in conjunction with significant antigenic variation among equine RVA genotypes, leading to limited cross-neutralization [48,121,141,240,241,242]. Also, the composition of the vaccines available in terms of the amount of virus and type of adjuvant included in the formulation still need to be harmonized, as has been done for cattle [243]. Recent studies, both in the field and in a suckling mouse model have demonstrated that the antibody response elicited against the G3P[12] vaccine strains marginally neutralize G14P[12] equine strains [48,244]. Limited vaccine efficacy has also been identified in the field among practitioners that routinely vaccinate pregnant mares to prevent rotaviral diarrhea (personal communication). Thus, exploration of modern vector platforms is critically needed to improve equine RVA vaccines. Additionally, with the likely emergence of equine RVB, current vaccines will potentially require the incorporation of equine RVB-specific antigens into their formulation in the future.

## 8. Diagnosis and Surveillance of RVA

In both humans and animals, RVA infections must be differentiated from other viral, bacterial, or parasitic causes of acute gastroenteritis. Additionally, and depending on the animal species and age range, RVA must be differentiated from other rotavirus species using laboratory testing. Etiologic diagnosis is performed on stool samples collected and kept refrigerated until testing is performed. While there are several methods available for the rapid direct detection of RVA (e.g., antigen tests such as enzyme-linked immunosorbent assays (ELISA) or lateral immunochromatographic methods, negative staining coupled with transmission electron microscopy, or a reverse-transcription polymerase chain reaction (RT-PCR)), rapid antigen tests are typically implemented in clinical settings, particularly immunochromatographic methods [245,246,247]. While the window for the detection of viral shedding using antigen-based detection methods is relatively short (up to one week after onset of diarrhea), viral nucleic acids can be detected for longer periods (e.g., via RT-PCR) [248]. Currently, multiplex real-time quantitative RT-PCR assays for the simultaneous detection of different agents associated with acute gastroenteritis including RVA have been widely implemented [249,250,251].

In contrast to routine diagnostic approaches, current surveillance of RVA heavily relies on molecular tools, primarily sequencing including next-generation sequencing (NGS) technologies. While initially characterized via serotyping and, subsequently, via genotyping of few genomic segments (i.e., that encoding for VP7 and VP4, determining G- and P-types), the emergence of NGS technologies has made surveillance and molecular epidemiology studies much more comprehensive, allowing for evaluation and comparison of full genome constellations of RVA strains from all around the globe. More rapid genotyping tools based on multiplex real-time RT-PCR have been developed and have become available for human RVA and equine RVA [252,253,254,255,256,257,258] but not for other animal RVA. These tools can be useful for rapid genotypification and further development for other animal species and are desired to contribute to monitoring.

Surveillance efforts of RVA in both humans and animals are critical to global health under the One Health concept, as animal RVAs contribute significantly to human RVA diversity and can lead to the emergence or re-emergence of reassortants with increased fitness and ability to spread in the human population. Additionally, surveillance is critical to assess vaccine coverage/efficacy, to determine selective pressures that can be exerted by current vaccines in both humans as well as livestock species, and to inform changes required in vaccine composition.

Since 2008, the WHO has coordinated the Global Rotavirus Surveillance Network (GRSN), a standardized global network of sentinel surveillance hospitals/laboratories (Global Rotavirus Laboratory Network (GRLN)) that report on clinical features and testing data to the WHO for children aged 5 years or less with acute gastroenteritis, and its weighting process ensures that each country is represented adequately within regional and global estimates. The overall goals include the follows: (1) Generate local data for decision making regarding rotavirus vaccine introduction and sustained use; (2) Assess and monitor disease trends and genotype distribution over time; (3) Develop a platform for vaccine effectiveness studies; (4) Highlight the value of surveillance data and use in fundraising and advocacy [259]. The performance of sentinel hospitals is regularly evaluated through WHO quality assurance programs [111]. Data derived from this surveillance program are primarily used for decision making in support of national vaccination strategies: (1) Evaluation of vaccine impact by assessing changes in rotavirus burden before and after vaccine introduction; (2) Evaluation of vaccine effectiveness [260]. In the last report from the GRSN, it has been determined that changes in circulating RVA genotypes do not seem to have diminished the overall impact of vaccinations globally; however, these dynamic changes in the predominance of RVA genotypes stress the importance of continued and sustained surveillance programs.

## 9. Conclusions

In conclusion, interspecies transmission and reassortment has significantly shaped the dynamics of rotavirus strains infecting and circulating in the human population, and animal-derived rotaviruses have demonstrated significant contribution to rotavirus diversity. Such transmission events and emergence of novel strains are important limiting factors on vaccine efficacy. Whether novel vaccine platforms/strategies could potentially assist in restricting viral pool evolution remains to be seen in the near future.

The relevance of ERVA as a potential zoonotic rotavirus needs to be considered under the One Health concept and based on the evidence available regarding interspecies transmission of RVA. Currently, efforts to undertake surveillance studies are insufficient and mostly limited to partial genomic sequencing typically restricted to VP7 and VP4. There is a need to establish comprehensive molecular surveillance programs of ERVA around the world using next-generation sequencing tools in order to work with whole genome data and better understand potential transmission dynamics or the contribution of genetic diversity to human RVA strains.

## Figures and Tables

**Table 1:** Genome organization, protein assignments, protein function, and location within the rotaviral particle.

Genome Segment	Encoded Protein	Genotype Acronym	Genotype	Size (bp) RVA-SA11 ^1^	Size (bp) ERVA-H2 ^2^	Size (aa, kDa) ^1^*	Virion Component	Function
1	VP1	R	RdRp	3302	3302	1088, 125	Core	RNA-dependent RNA polymerase
2	VP2	C	Core protein	2693	2687	881 (880), 102.4	Core	Core shell protein
3	VP3	M	Methyltransferase	2591	2591	835, 98.1	Core	Capping enzyme
4	VP4	P	Protease-sensitive	2362	2362	776, 86.8	Outer layer	Spike protein
5	NSP1	A	Interferon antagonist	1614	1579	496 (498), 58.7	-	Interferon antagonist, putative viral E3 ubiquitin ligase
6	VP6	I	Inner capsid	1356	1356	397, 44.8	Middle layer	Intermediate capsid protein
7	NSP3	T	Translation enhancer	1105	1066	315 (313), 34.6	-	Binding of 3′ viral mRNA and eukaryotic initiation factor eIF4G
8	NSP2	N	NTPase	1059	1059	317, 36.7	-	Viroplasm matrix protein with NTPase
9	VP7	G	Glycosylated	1062	1059	326, 37.4	Outer layer	Surface glycoprotein
10	NSP4	E	Enterotoxin	751	752	175, 20.3	-	Essential for budding into ER; enterotoxin
11	NSP5	H	Phosphoprotein	667	666	198, 21.7	-	Viroplasm component with RNA binding activity and interacting with NSP2
11	NSP6	-	-	279	267	92 (88), 12	-	Viroplasm component interacting with NSP5

^1^ Genome segment and protein sizes based on GenBank Accession numbers NC_011500–NC_011510. ^2^ Genome segment and protein sizes based on GenBank Accession numbers KM454492.1–KM454502.1. * If number of amino acids differs between RVA-SA11 and ERVA-H2, the number of amino acids for ERVA-H2 are shown in parenthesis.

**Table 2:** Genetic relationships among selected RVA strains occurring in humans and animals with a specific emphasis on those of animal origin. The colors green, red, and orange represent Wa-like, DS-1-like, and AU-1-like gene segments. SA-11-like gene segments are depicted in blue. Genome constellation designation is based on the proposed nomenclature (Gx-P[x]-Ix-Rx-Cx-Mx-Ax-Nx-Tx-Ex, where x is an Arabic number. Genogroups were assigned based on the available literature. Equine-like G3 strains are highlighted using an asterisk. The table was modified from Matthijnssens et al., 2008 [46], Matthijnssens et al., 2012 [54], and Martella et al., 2010 [2].

Strain	Host	Genogroup	VP7	VP4	VP6	VP1	VP2	VP3	NSP1	NSP2	NSP3	NSP4	NSP5
Wa	Human	Wa-like (1)	G1	P[8]	I1	R1	C1	M1	A1	N1	T1	E1	H1
KU	Human	Wa-like (1)	G1	P[8]	I1	R1	C1	M1	A1	N1	T1	E1	H1
Dhaka16-03	Human	Wa-like (1)	G1	P[8]	I1	R1	C1	M1	A1	N1	T1	E1	H1
P(rice)	Human	Wa-like (1)	G3	P[8]	I1	R1	C1	M1	A1	N1	T1	E1	H1
WI61	Human	Wa-like (1)	G9	P[8]	I1	R1	C1	M1	A1	N1	T1	E1	H1
B4633-03	Human	Wa-like (1)	G12	P[8]	I1	R1	C1	M1	A1	N1	T1	E1	H1
Gottfried	Porcine	Wa-like (1)	G4	P[6]	I1	R1	C1	M1	A8	N1	T1	E1	H1
A131	Porcine	Wa-like (1)	G3	P[7]	I5	R1	C2	M1	A1	N1	T1	E1	H1
OSU	Porcine	Wa-like (1)	G5	P[7]	I5	R1	C1	M1	A1	N1	T1	E1	H1
A253	Porcine	Wa-like (1)	G11	P[7]	I5	R1	C2	M1	A1	N1	T1	E1	H1
YM	Porcine	Wa-like (1)	G11	P[7]	I5	R1	C1	M1	A8	N1	T1	E1	H1
DS-1	Human	DS-1-like (2)	G2	P[4]	I2	R2	C2	M2	A2	N2	T2	E2	H2
TB-Chen	Human	DS-1-like (2)	G2	P[4]	I2	R2	C2	M2	A2	N2	T2	E2	H2
DRC86	Human	DS-1-like (2)	G8	P[6]	I2	R2	C2	M2	A2	N2	T2	E2	H2
N26-02	Human	DS-1-like (2)	G12	P[6]	I2	R2	C2	M2	A2	N1	T2	E6	H2
S13-30 *	Human	DS-1-like (2)	G3	P[4]	I2	R2	C2	M2	A2	N2	T2	E2	H2
S13-45 *	Human	DS-1-like (2)	G3	P[4]	I2	R2	C2	M2	A2	N2	T2	E2	H2
IS1078/IS1090/MI1125 *	Human	DS-1-like (2)	G3	P[8]	I2	R2	C2	M2	A2	N2	T2	E2	H2
AM-16-31 *	Human	DS-1-like (2)	G3	P[8]	I2	R2	C2	M2	A2	N1	T2	E2	H2
SOEP128 *	Human	DS-1-like (2)	G3	P[6]	I2	R2	C2	M2	A2	N1	T2	E2	H2
Hun5	Human	DS-1-like (2)	G6	P[14]	I2	R2	C2	M2	A11	N2	T6	E2	H3
MG6	Human	DS-1-like (2)	G6	P[14]	I2	R2	C2	M2	A11	N2	T6	E2	H3
PA169	Human	DS-1-like (2)	G6	P[14]	I2	R2	C2	M2	A3	N2	T6	E2	H3
111/05-27	Human	DS-1-like (2)	G6	P[14]	I2	R2	C2	M2	A3	N2	T6	E2	H3
H2	Horse		G3	P[12]	I6	R2	C2	M3	A10	N2	T3	E2	H7
E30	Horse		G3	P[12]	I6	R2	C2	M3	A10	N2	T3	E12	H7
03V04954	Horse		G3	P[12]	I6	R2	C2	M3	A10	N2	T3	E2	H7
E403	Horse		G14	P[12]	I2	R2	C2	M3	A10	N2	T3	E12	H7
E4040	Horse		G14	P[12]	I2	R2	C2	M3	A10	N2	T3	E12	H7
FI23	Horse		G14	P[12]	I2	R2	C2	M3	A10	N2	T3	E12	H7
NCDV-Lincoln	Bovine	DS-1-like (2)	G6	P[1]	I2	R2	C2	M2		N2	T6	E2	
BRV033	Bovine	DS-1-like (2)	G6	P[1]	I2	R2	C2	M2	A3	N2	T6	E2	H3
UK	Bovine	DS-1-like (2)	G6	P[5]	I2	R2	C2	M2	A3	N2	T7	E2	H3
WC3	Bovine	DS-1-like (2)	G6	P[5]	I2	R2	C2	M2	A3	N2	T6	E2	H3
AU-1	Human	AU-1-like (3)	G3	P[9]	I3	R3	C3	M3	V3	N3	T3	E3	H3
T152	Human	AU-1-like (3)	G12	P[9]	I3	R3	C3	M3	A12	N3	T3	E3	H6
B4106	Human		G3	P[14]	I2	R2	C2	M3	A9	N2	T6	E5	H3
30/96	Lapine		G3	P[14]	I2	R2	C2	M3	A9	N2	T6	E5	H3
Cat97	Feline		G3	P[3]	I3	R3	C2	M3	A9	N2	T3	E3	H6
CU-1	Canine		G3	P[3]	I3	R3	C2	M3	A9	N2	T3	E3	H6
K9	Canine		G3	P[3]	I3	R3	C2	M3	A9	N2	T3	E3	H6
Ro1845	Human		G3	P[3]	I3	R3	C2	M3	A9	N2	T3	E3	H6
HRC3	Human		G3	P[3]	I3	R3	C2	M3	A9	N2	T3	E3	H6
SA11-5S	Simian		G3	P[1]	I2	R2	C5	M5	A5	N5	T5	E2	H5

* Equine-like G3 strains. This is only a small representation of several strains reported (Komoto et al., 2018 [55], Katz et al., 2019 [56], Utsumi et al., 2018 [57], Guerra et al., 2016 [58], and other references thereof).

## Data Availability

Not applicable.
